# Factors associated with institutional delivery in Ghana: the role of decision-making autonomy and community norms

**DOI:** 10.1186/s12884-014-0398-7

**Published:** 2014-11-27

**Authors:** Ilene S Speizer, William T Story, Kavita Singh

**Affiliations:** Carolina Population Center, University of North Carolina at Chapel Hill, Chapel Hill, NC USA; Department of Maternal and Child Health, Gillings School of Global Public Health, University of North Carolina at Chapel Hill, Chapel Hill, NC USA

**Keywords:** Institutional delivery, Ghana, Maternal health, Autonomy, Social support

## Abstract

**Background:**

In Ghana, the site of this study, the maternal mortality ratio and under-five mortality rate remain high indicating the need to focus on maternal and child health programming. Ghana has high use of antenatal care (95%) but sub-optimum levels of institutional delivery (about 57%). Numerous barriers to institutional delivery exist including financial, physical, cognitive, organizational, and psychological and social. This study examines the psychological and social barriers to institutional delivery, namely women’s decision-making autonomy and their perceptions about social support for institutional delivery in their community.

**Methods:**

This study uses cross-sectional data collected for the evaluation of the Maternal and Newborn Referrals Project of Project Fives Alive in Northern and Central districts of Ghana. In 2012 and 2013, a total of 2,527 women aged 15 to 49 were surveyed at baseline and midterm (half in 2012 and half in 2013). The analysis sample of 1,606 includes all women who had a birth three years prior to the survey date and who had no missing data. To determine the relationship between institutional delivery and the two key social barriers—women’s decision-making autonomy and community perceptions of institutional delivery—we used multi-level logistic regression models, including cross-level interactions between community-level attitudes and individual-level autonomy. All analyses control for the clustered survey design by including robust standard errors in Stata 13 statistical software.

**Results:**

The findings show that women who are more autonomous and who perceive positive attitudes toward facility delivery (among women, men and mothers-in-law) were more likely to deliver in a facility. Moreover, the interactions between autonomy and community-level perceptions of institutional delivery among men and mothers-in-law were significant, such that the effect of decision-making autonomy is more important for women who live in communities that are less supportive of institutional delivery compared to communities that are more supportive.

**Conclusions:**

This study builds upon prior work by using indicators that provide a more direct assessment of perceived community norms and women’s decision-making autonomy. The findings lead to programmatic recommendations that go beyond individuals and engaging the broader network of people (husbands and mothers-in-law) that influence delivery behaviors.

## Background

As we approach 2015 and the deadline for attainment of the Millennium Development Goals (MDG), increased attention and effort are being given to reaching target populations with specific programmatic strategies, especially in countries that are not on track to attain the MDGs. In Ghana, the site of this study, where the maternal mortality ratio (MMR) remains high at 350 maternal deaths per 100,000 live births [1] and under-five mortality is estimated at 82 deaths per 1000 live births [2], there has been increased attention to initiatives to improve maternal, infant, and child health services. To help in the attainment of improved infant and child health (MDG 4) and improved maternal health (MDG 5), programs in Ghana and elsewhere promote skilled attendance at delivery; in many low income countries, this is equated with institutional (also called facility) delivery [3]. Skilled attendance at delivery means having an accredited health professional, including a midwife, doctor, or nurse, who has been trained in the skills needed to manage a normal or uncomplicated pregnancy and childbirth and to support the woman in the immediate postpartum period. This person should also be able to identify, manage and refer complications experienced by the woman or the newborn [4].

Numerous studies have demonstrated that in sub-Saharan Africa, including Ghana, there is often high use of antenatal care services but lower use of institutional delivery [2,5,6]. For example, the 2008 Ghana Demographic and Health Survey (GDHS) showed that more than 95% of women who had a birth in the last five years received antenatal care from a skilled provider prior to birth [6]. As compared to high antenatal care use, only 57% of women had an institutional delivery and only 59% delivered with a skilled attendant present [6]; similar distinctions are found in the 2011 Multiple Indicator Cluster Survey [2]. Wide variability in institutional delivery and skilled attendance at delivery was observed by region of residence with the Northern region having the lowest percentage of women delivering in a facility and the Central region falling in the middle on percentage of births in a facility [6]; these are the two regions covered in this study.

A number of studies using the GDHS demonstrated important demographic- and policy-level factors associated with institutional delivery [7-9]. Recent global studies have examined common barriers to antenatal care and institutional delivery [3,10-17]. Much of this research has focused on transportation, distance, and cost [13,15,16]. A recent qualitative study by Matsuoka and colleagues in Cambodia (2010) demonstrated five types of barriers to utilization of government maternal health services [17]. These barriers were: financial; physical; cognitive; organizational; and psychological and social. Often, the financial and physical barriers are examined together to capture issues around transportation and distance to a facility as well as costs to reach or use the facility [13,15-17]. Ghana has implemented the National Health Insurance Scheme in an effort to reduce these types of financial barriers. Recent studies from Ghana have found that women with health insurance were more likely to have an institutional delivery [18-20] and insurance was associated with better maternal and child health outcomes [20]. Cognitive barriers are related to misconceptions about services offered and concerns about quality of services [17]. Organizational barriers are focused on the role of the providers in terms of attitudes, availability, and services offered [17,21]; these have been found to be particularly important from qualitative studies in Ghana [14,21]. Finally, psychological and social barriers are related to community norms and attitudes toward facility delivery and toward the staff at the facility [3,17]. These barriers to health care have been demonstrated in various cultural contexts including Bangladesh [22], Cambodia [17], Nepal [23], rural Kenya [13], and Northern Ghana [10] mostly using qualitative data or nationally representative data from Demographic and Health Surveys.

Social barriers to institutional delivery, such as community attitudes towards institutional delivery and levels of decision-making autonomy among women, have received much less attention in the literature [24]. Community beliefs and attitudes about maternal health behaviors have been shown to influence a woman’s individual decision to seek care. For example, in a study of six countries in sub-Saharan Africa, Stephenson and colleagues [25] found that community norms about facility-based delivery and women’s decision-making autonomy were potential pathways that influenced the decision to deliver a child in a health facility. In rural Tanzania, community beliefs that facility delivery is important for the health of the mother and baby were associated with use of facility-based delivery [26]. In a separate study among the same population in Tanzania, male partners’ opinions about institutional delivery were associated with actual institutional delivery, such that spouses who disagreed about the importance of institutional delivery were less likely to have one compared to spouses who agreed that delivering in a health facility was important [27]. In rural Mali, mothers-in-law’s beliefs and attitudes were demonstrated to have an influence on their daughters-in-law’s maternal health care-seeking behaviors [28].

Previous studies have also shown that women’s decision-making autonomy is associated with the use of health facilities for delivery. In Nigeria, women with greater decision-making autonomy were more likely to deliver in a health facility, which may indicate that these women were better able to advocate for and access a health facility for childbirth [29]. In Bangladesh, households in which husbands made decisions alone were associated with less use of antenatal care and skilled delivery care compared to households that practiced joint decision-making [30]. The relationship between women’s decision-making autonomy and use of maternal health services may be due to women’s power to realize their preferences, which includes a stronger preference for ensuring their own health [31]. Although some studies have demonstrated the importance of community norms and women’s decision-making autonomy on the decision to deliver in a health facility, there have been few studies that have looked at the two pathways together and examined the ways in which community norms and household decision-making autonomy interact with one another.

This study contributes to our understanding of autonomy and social barriers to institutional delivery in Ghana using recently collected quantitative data from two regions of Ghana. Because the focus of the study was to obtain information on barriers to institutional delivery and women’s referral experiences, this study includes a large sample of women who recently delivered a child, providing rich information on barriers to institutional delivery in these regions. The objectives of this study are to examine whether women’s decision-making roles and their perceptions about social support for facility delivery, measured at the individual and community levels, are associated with women’s actual place of delivery in Ghana. Furthermore, we examine how the relationship between community-level attitudes and institutional delivery differs for households in which women have a say in their own health care and those that do not.

## Methods

### Data collection

The cross-sectional data for this study come from baseline and midline surveys that were used during the evaluation of the Maternal and Newborn Referrals Project of Project Fives Alive in the Northern and Central Regions of Ghana. The Maternal and Newborn Referrals project is being implemented by the Institute for Healthcare Improvement (IHI), the National Catholic Health Service (NCHS) and the Ghana Health Service (GHS). The evaluation is being led by the University of North Carolina at Chapel Hill and the University of Ghana. Baseline data were collected between May and June 2012 to help design the project and midline data were collected between October and November 2013 to help strengthen project implementation. Since the focus of this study is not significantly affected by the interventions implemented as part of the Maternal and Newborn Referrals Project and there were delays in project initiation until July, 2013, the baseline and midline data were merged to provide a larger cross-sectional sample.

Multiple survey instruments were used at baseline and midline, including a household survey, a community leader survey, and facility surveys (with clients, providers, traditional birth attendants, and chemical sellers). This analysis focuses on the household survey data. The purpose of the household survey was to obtain information on knowledge, attitudes and practices regarding maternal and child health services.

The household survey used the 30-by-N cluster sample design; this method is commonly used in child survival programs [25,32]. Cluster sampling is an efficient sampling method because it provides a means to obtain a representative sample from the region without undertaking a census of households in the community. However, cluster sampling leads to biased standard errors due to the correlation between observations from the same cluster. We explain our approach for accounting for the biased standard errors in the analysis section. The overall sampling strategy was designed to meet the evaluation objectives for the Maternal and Newborn Referrals Project [18]. At baseline, the goal was to include a large sample of recently pregnant women (pregnant in the last 12 months) to identify their experiences with pregnancy, childbirth, and newborn health. Thus, we used a 30-by-7 sampling approach to identify thirty clusters per region (thirty from the three districts in the Northern region and thirty from the three districts in the Central region), and seven recently pregnant women in each cluster were randomly selected for interview. Random selection of clusters was undertaken from an exhaustive list of communities in the six study districts. The recently pregnant women were randomly sampled from a list of all recently pregnant women in the community (determined through interviews with community leaders and health workers in the community). To supplement the sample of 210 recently pregnant women per region, we also included 14 nearby neighbor women (ages 15–49) who may or may not have been recently pregnant to permit an examination of maternal and newborn health knowledge, attitudes, and behaviors of women in the community. At midline, the same 30-by-N cluster design was employed, however, a new sample of communities was drawn from the same districts. As with the baseline survey, in all selected clusters, seven recently pregnant women were surveyed as well as 14 nearby neighbors.

For the purpose of this study, which uses baseline and midline data, and accounting for plausible design effect, our sample size is adequate to obtain precise estimates of our key outcome (institutional delivery). A total of 2,527 women were interviewed in the two rounds of data collection (1,267 women were interviewed at baseline and a new sample of 1,260 women were interviewed at midline). This analysis of institutional delivery focuses on a sub-sample of the 2,527 women interviewed, which excludes women who did not have a birth in the last three years, were not in union, or had missing information on the key variables of interest. Thus the final analysis sample is 1,606 women.

Ethics review approval for the study was obtained by the University of North Carolina at Chapel Hill and the Ghana Health Service. Informed consent was obtained from all study participants.

### Variables

The key dependent variable for this analysis is the place of delivery of the last birth in the last three years. Women who delivered in a health facility are coded one, whereas all women who delivered at home or in the home of someone else (e.g., a relative or a health worker) are coded zero (i.e., non-institutional delivery).

The main independent variables for this analysis focus on decision-making autonomy and attitudes toward institutional delivery. First, all women were asked: “Who usually makes decisions about health care for you?” Women who reported that they make the decisions alone or make the decisions jointly with their partner were the reference group (high decision-making autonomy), and were compared to women who reported that their partner makes the decision alone (low decision-making autonomy). A third category was also created for the small number of women who reported that someone else makes the decision. The other independent variables of interest are attitudes toward institutional delivery, represented by three separate questions. First, all women were asked: “How many women do you think in your community deliver their baby in a health facility?” Response options were: none, some, most, and all (coded 1–4); the small number of women who reported “don’t know” (n = 114) were dropped from the analysis. Second, women were asked: “In your opinion, what percentage of men in your community is supportive of facility delivery?” Response options were: no men, few men, some men, most men, and all men (coded 1–5); the 149 women who reported “don’t know” were dropped from the analysis. Finally women were asked: “In your opinion, what percentage of mothers-in-law in your community is supportive of facility delivery?” Response options were: no mothers-in-law, some mothers-in-law, most mothers-in-law, and all mothers-in-law (coded 1–4); the 172 women who reported “don’t know” were dropped from the analysis.

To examine community-level attitudes toward facility delivery, we also created comparable variables at the community-level for each of the three attitude questions. In particular, for each woman, we calculated the average response on how many women in the community she perceived had delivered their baby in a health facility. These community-level responses were calculated by creating an average value of all women living in the cluster, removing each individual woman from the calculation. A similar approach was undertaken for the community-level men’s attitude and the community-level mother-in-law’s attitude.

All models control for demographic factors previously found to be associated with facility delivery including age, education, ethnicity, employment status, religion, parity, wealth, region, and time period (baseline or midline) [24]. See Table 1 for a description of these variables. The wealth variable was created based on three household characteristics: type of toilet, type of fuel used in the household, and location of the kitchen. Households with a non-improved toilet facility (as defined in the Ghana DHS), that use wood for their source of fuel, and that have a kitchen outside their household were coded as being the poorest households. Households with two out of three of these lower quality scenarios were considered to be medium, and households with none or just one of these lower quality scenarios were considered to be the richest. This is the same approach that was used in an earlier analysis of health insurance effects on facility delivery using these same data [18]. In the full sample, based on this classification, about 40% of the women were in the poorest category, 40% in the medium category, and only 19% were in the richest category (see Table 1). It is worth noting that use of antenatal care (ANC) during the pregnancy was not included as an independent variable in the reduced form models presented. Previous research has demonstrated that use of ANC is endogenous and would introduce bias into the models presented [33].

**Table 1:** **Characteristics of total sample (baseline and midline), recent birth sample, and analysis sample from Ghana evaluation of Maternal and Newborn Referrals Project, 2012, 2013**

**Characteristics**	**Full sample**		**Recent birth sample (birth in the last 3 years)**		**Analysis sample (in union, birth in the last 3 years, no missing information)**	
	**Percent**	Number (n = 2527*)	**Percent**	Number (n = 1840*)	**Percent**	**Number (n = 1606)**
Age:						
<19	7.8	196	7.5	138	4.7	76
20-24	23.7	597	25.4	468	23.4	375
25-34	44.7	1,127	48.7	896	51.9	833
35-49	23.9	604	18.4	338	20.1	322
Education:						
None	47.4	1,199	45.3	835	50.0	803
Primary	20.1	508	20.5	378	18.4	295
Secondary or higher	32.4	819	34.2	629	31.6	508
Ethnicity:						
Akan	47.9	1,210	50.3	926	45.6	732
Mole-Dagbani	32.1	811	30.8	568	34.5	554
Konkomba	9.6	242	8.9	164	9.3	150
Other	10.4	264	10.0	184	10.6	170
Work status:						
Unpaid/unemployed	58.3	1,474	59.3	1,092	57.7	927
Self employed	38.1	964	37.1	684	38.7	622
Paid work	3.5	89	3.6	66	3.6	57
Religion:						
Christian	56.0	1,414	58.1	1,071	54.4	874
Muslim	29.8	752	28.1	517	30.7	493
None/traditional/other	14.3	361	13.8	254	14.9	239
Marital status:						
Not currently in union	15.1	381	12.7	234	0.0	0
Currently in union	84.9	2,146	87.3	1,608	100.0	1606
Parity:						
0	4.9	124	0.0	0	0	0
1	20.8	526	23.2	427	18.4	296
2	15.7	397	17.9	329	17.3	277
3	14.0	352	14.9	275	16.2	260
4	12.7	322	13.1	242	14.2	228
5	9.6	242	9.9	183	10.9	175
6+	22.3	564	21.0	386	23.0	370
Region:						
Central	50.1	1,267	52.6	968	48.0	770
Northern	49.9	1,260	47.5	874	52.1	836
Wealth category						
Poorest	40.4	1,022	40.5	745	41.5	667
Medium	40.3	1,019	40.3	742	39.3	631
Richest	19.2	486	19.3	355	19.2	308
Time						
Baseline	50.1	1,267	45.7	841	45.8	735
Midline	49.9	1,260	54.3	1011	54.2	871

*The sample size is slightly smaller for some variables that had missing data.

### Analysis

We use bivariate analyses to examine the association between the key independent variables and institutional delivery, controlling for key demographic variables described earlier and adjusting for the clustered survey design. Because the dependent variable of interest (institutional delivery) is binary, and we are interested in both individual and community-level attitudes, we use multi-level logistic regression models. To examine if the relationship between community-level attitudes and institutional delivery differs by women’s decision-making autonomy, we use models with cross-level interactions between community-level attitudes and individual-level autonomy. Since the interactions cannot be evaluated by looking at the sign, magnitude, or statistical significance of the odds ratio for nonlinear models [34], we plot the interaction using the *margins* command in Stata 13. All regression analyses adjust for the clustered survey design by including robust standard errors in Stata 13 statistical software. Regression results are presented by showing the odds ratios and the 95% confidence intervals.

## Results

Table 1 presents the descriptive characteristics of three sample populations: the full sample (n = 2,527), the sample of women who had a recent birth (n = 1,840), and the final analysis sample of women in union who had a recent birth and had non-missing information on all study variables (n = 1,606). Women who were not in union were dropped from the analysis since one of the key independent variables on autonomy was specifically about decision-making in union. The only difference observed between the full sample and the final analysis sample is that the analysis sample is made up of a greater percentage of women from the midline sample. At midline, the study recruited a greater number of recently pregnant women in the neighbor sample, increasing the proportion of the sample from midline in the recently pregnant sample. More than two-fifths of the women surveyed for this study are uneducated, and more than two-fifths are of Akan and one-third is of Mole-Dagbani ethnicity. More than half of the women are unemployed or doing unpaid work. More than half of the sample is Christian, and a little less than a third is Muslim. In the analysis sample, all women have had at least one birth (since the outcome is place of delivery of the last birth) with about equal proportions having one, two, or three births. Twenty-three percent of the women have had six or more births. Finally, the full sample is evenly divided between the Central and Northern regions, whereas the analysis sample includes a greater percentage of women from the Northern region; this may be suggestive of more pregnancies among women in union in the Northern region. To assess if differences between the baseline and midline samples are influencing the results, we re-ran the regression analyses with only the baseline sample; the results were similar for the key independent variables of interest (results not shown).

Table 2 presents the distribution of the key independent variables (perceptions about facility delivery and decision-making autonomy) in the analysis sample and then by whether or not the woman had a facility delivery. First, women’s attitudes toward how many women deliver in a health facility indicate that more than half of women report that most women deliver in a health facility (52%), and another 13% report that all women deliver in a facility. About 35% of women report none or some women deliver in a facility. Second, women’s perceptions of men’s opinions of facility delivery indicate that the majority of women (about 70%) believe that most men or all men are supportive of facility delivery. About 30% of women report that no men, few men and some men are supportive of facility delivery. Third, women’s perceptions of mothers-in-law’s opinions are similar to women’s perceptions of men’s opinions of facility delivery. Fourth, about half of the women report that they alone, or with their partner, make decisions about their own health care. Forty-seven percent of women report that their husband alone makes these health care decisions and only 4% report that someone else makes these decisions. When comparing the attitude and autonomy variables by whether or not the woman had a facility delivery, a significant difference is found in the expected direction, such that those women who had a facility delivery have attitudes that are more supportive of facility delivery than those women who did not have a facility delivery.

**Table 2:** **Attitudes toward facility delivery and who makes decisions in the household and distribution by whether the woman had a facility delivery in Ghana 2012, 2013**

**Characteristics**	Analysis sample (in union with recent birth)*		**Distribution by whether had a facility delivery for last birth**	
	**Percent**	**Number**	**Non-facility delivery (47.0%)**	**Facility delivery (53.0%)**
How many women do you think in your community deliver their baby in a health facility?				
None	5.7	87	10.7	1.3
Some	29.1	446	40.1	19.7
Most	52.4	803	43.4	60.1
All	12.9	197	5.8	18.9***
In your opinion, what percentage of men in your community are supportive of facility delivery?				
No men	1.9	29	4.0	0.1
Few men	11.5	174	17.7	6.2
Some men	17.0	257	20.8	13.7
Most men	52.8	797	46.9	57.9
All men	16.8	253	10.6	22.1***
In your opinion, what percentage of mother-in-laws in your community are supportive of facility delivery?				
No mothers-in-law	3.9	59	7.6	0.8
Some mothers-in-law	29.8	446	37.4	23.2
Most mothers-in-law	51.2	767	44.0	57.4
All mothers-in-law	15.1	227	11.1	18.7***
Who usually makes decisions about health care for you:				
Woman alone/both partners	49.3	791	39.9	57.5
Husband only	46.6	748	57.7	36.7
Other	4.2	67	2.4	5.8***

*Note, those who reported “don’t know” to the attitude questions were dropped, this was 114 for the question on community attitudes; 149 for the question on men’s attitudes; and 172 for the question on mother-in-law attitudes. Significance testing compares facility delivery to non-facility delivery. ***F-test p value ≤0.001.

Table 3 presents the odds ratios and 95% confidence intervals for the analysis of having a facility delivery compared to not having a facility delivery among women in union who had a birth in the last three years. The key variables of interest to this analysis are at the bottom of the table. Note that because of high correlation between the attitude variables (e.g., attitudes about facility delivery for all women, attitudes about men’s support for facility delivery and attitudes about mother-in-law’s support for facility delivery), models were run separately for each of these independent variables.

**Table 3:** **Logistic regression odds ratios and 95% confidence intervals of association between attitudes and decision-making autonomy and whether a woman delivered a recent birth in a facility, Ghana, 2012, 2013**

**Characteristics**	**Facility delivery vs. non-facility delivery**	**Facility delivery vs. non-facility delivery**	**Facility delivery vs. non-facility delivery**	**Facility delivery vs. non-facility delivery**	**Facility delivery vs. non-facility delivery**	**Facility delivery vs. non-facility delivery**
	**Model 1**	**Model 2**	**Model 3**	**Model 4**	**Model 5**	**Model 6**
Time						
Baseline	1.0	1.0	1.0	1.0	1.0	1.0
Midline	1.20 (0.80-1.81)	1.21 (0.81-1.81)	0.83 (0.56-1.25)	0.85 (0.57-1.27)	0.84 (0.55-1.28)	0.86 (0.56-1.32)
Age:						
<25	1.0	1.0	1.0	1.0	1.0	1.0
25-34	1.00 (0.71-1.39)	1.00 (0.71-1.40)	1.06 (0.77-1.46)	1.07 (0.77-1.47)	1.00 (0.73-1.37)	1.00 (0.73-1.38)
35-49	1.17 (0.74-1.85)	1.18 (0.74-1.86)	1.24 (0.79-1.93)	1.26 (0.81-1.98)	1.13 (0.75-1.71)	1.15 (0.76-1.74)
Education:						
None	1.0	1.0	1.0	1.0	1.0	1.0
Primary	1.40 (0.94-2.09)+	1.41 (0.94-2.10)+	1.31 (0.92-1.88)	1.32 (0.92-1.90)	1.43 (1.01-2.03)*	1.45 (1.02-2.07)*
Secondary or higher	1.92 (1.27-2.92)**	1.93 (1.27-2.92)**	2.04 (1.40-2.96)***	2.06 (1.42-2.99)***	2.21 (1.52-3.23)***	2.26 (1.55-3.30)***
Ethnicity:						
Akan	1.0	1.0	1.0	1.0	1.0	1.0
Mole-Dagbani	1.43 (0.43-4.84)	1.40 (0.41-4.72)	1.25 (0.34-4.61)	1.15 (0.32-4.20)	1.51 (0.45-5.09)	1.47 (0.44-4.93)
Konkomba	5.31 (1.69-16.69)**	5.43 (1.74-16.96)**	3.25 (0.97-10.88)+	3.33 (1.01-10.95)*	2.34 (0.74-7.36)	2.40 (0.77-7.46)
Other	1.45 (0.60-3.51)	1.45 (0.60-3.48)	1.35 (0.56-3.27)	1.30 (0.55-3.12)	1.49 (0.67-3.31)	1.46 (0.66-3.24)
Work status:						
Unpaid/unemployed	0.75 (0.57-1.00)+	0.76 (0.57-1.01)+	0.80 (0.61-1.06)	0.81 (0.62-1.07)	0.72 (0.55-0.95)*	0.73 (0.56-0.96)*
Self-employed/paid work	1.0	1.0	1.0	1.0	1.0	1.0
Religion:						
Christian	1.0	1.0	1.0	1.0	1.0	1.0
Muslim	1.34 (0.67-2.66)	1.33 (0.67-2.63)	1.25 (0.57-2.77)	1.23 (0.57-2.70)	1.25 (0.58-2.72)	1.23 (0.57-2.64)
None/traditional/other	0.53 (0.33-0.86)**	0.53 (0.33-0.86)**	0.50 (0.30-0.83)**	0.51 (0.31-0.84)**	0.47 (0.30-0.75)**	0.47 (0.29-0.75)***
Parity (continuous):	0.95 (0.92-0.98)**	0.95 (0.92-0.98)**	0.95 (0.92-0.98)**	0.95 (0.92-0.98)**	0.96 (0.93-0.99)**	0.96 (0.93-0.99)**
Region:						
Central	2.83 (0.96-8.33)+	2.73 (0.92-8.09)+	1.98 (0.62-6.39)	1.84 (0.57-5.93)	2.15 (0.72-6.44)	2.06 (0.68-6.23)
Northern	1.0	1.0	1.0	1.0	1.0	1.0
Wealth category						
Poorest	1.0	1.0	1.0	1.0	1.0	1.0
Medium	1.30 (0.99-1.72)+	1.30 (0.98-1.72)+	1.24 (0.93-1.63)	1.21 (0.91-1.61)	1.28 (0.97-1.68)+	1.26 (0.96-1.67)
Richest	1.93 (1.32-2.82)***	1.93 (1.32-2.83)***	1.87 (1.25-2.81)**	1.87 (1.24-2.81)**	1.94 (1.29-2.90)***	1.95 (1.30-2.92)***
Decision-making about woman’s healthcare						
Woman/joint decision-making	1.0	1.0	1.0	1.0	1.0	1.0
Husband alone	0.81 (0.63-1.05)	0.37 (0.07-1.99)	0.74 (0.58-0.96)*	0.04 (0.01-0.32)**	0.73 (0.57-0.94)*	0.08 (0.01-0.50)**
Other	2.22 (1.09-4.53)*	5.73 (0.14-227.93)	1.65 (0.82-3.31)	0.41 (0.01-23.09)	1.77 (0.83-3.76)	1.11 (0.03-42.74)
Perceived number of women that delivery in facility	1.83 (1.53-2.19)***	1.84 (1.53-2.20)***	Na	Na	Na	Na
Perception of men’s attitude toward facility delivery	Na	Na	1.36 (1.18-1.55)***	1.36 (1.19-1.56)***	Na	Na
Perception of MIL attitude toward facility delivery	Na	Na	Na	Na	1.40 (1.18-1.65)***	1.41 (1.19-1.66)***
Community attitudes						
Attitudes toward number that delivery in facility	4.13 (2.49-6.85)***	3.62 (2.02-6.51)***	Na	Na	Na	Na
Men’s attitudes toward facility delivery	Na	Na	3.70 (2.27-6.05)***	2.48 (1.47-4.19)***	Na	Na
MIL attitudes toward facility delivery	Na	Na	Na	Na	3.46 (1.85-6.45)***	2.20 (1.11-4.34)*
Interactions						
Community attitude *Husband alone decides	Na	1.33 (0.72-2.45)	Na	2.14 (1.26-3.62)**	Na	2.25 (1.15-4.42)*
Community attitude *Other decide	Na	0.70 (0.19-2.59)	Na	1.45 (0.50-4.22)	Na	1.18 (0.30-4.62)

Na – not applicable for that model. Sample size slightly smaller than full sample since small number of missing observations were dropped for perception questions by model.

+p ≤ 0.10; *p ≤ 0.05; **p ≤ 0.01; ***p ≤ 0.001.

Model 1 demonstrates that women who perceive that a greater number of women in their community deliver in a facility are significantly more likely to have delivered their last birth in a facility (OR: 1.83; 95% CI: 1.53-2.19) compared to women who think fewer women deliver in a facility. Furthermore, women who report that someone other than herself or her spouse makes decisions about her own health care were more likely to deliver in a facility (OR: 2.22; 95% CI: 1.09-4.53) compared to women who are involved in the decision-making process. This variable is not significant in the other models and may merit consideration in future analyses. Community-level attitude toward facility delivery is also significant in Model 1 indicating that, controlling for the woman’s own attitude toward the number of women who deliver in a facility, women who live in communities where more women perceive higher use of facility delivery are more likely to deliver in a facility than women who live in communities where women perceive fewer facility deliveries (OR: 4.13; 95% CI: 2.49-6.85). The patterns for the key variables of interest remained the same in Model 2 and there was no significant interaction between decision-making autonomy and community-level attitudes.

**Figure 1:**
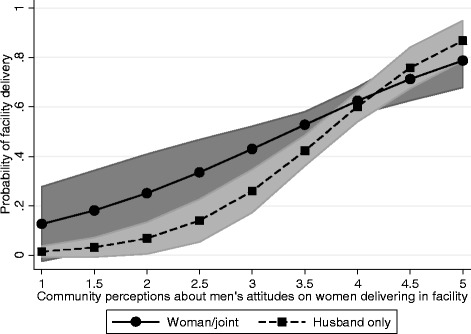
**Adjusted predictions at representative values with 95% confidence intervals for Model 4.** Community perceptions of husbands’ attitudes toward facility delivery by decision-making autonomy.

Models 3 and 4 tell a slightly different story. In Model 3, women who perceive that more men are supportive of facility delivery are significantly more likely to deliver in a facility (OR: 1.36; 95% CI: 1.18-1.55). In addition, women who report that the husband alone makes decisions about her health care are significantly less likely to have a facility delivery than women who report that she is involved in decision-making (OR: 0.74; 95% CI: 0.58-0.96). Model 3 also shows that controlling for women’s own attitudes toward men’s support for facility delivery, women who live in communities where men are perceived to have more positive attitudes are significantly more likely to have a facility delivery than all others (OR: 3.70; 95% CI: 2.27-6.05). Model 4, which adds an interaction term between women’s decision-making autonomy and men’s community-level attitudes, shows that the effect of the husband making the decision alone becomes more important (and remains negative), particularly for women with less male support for facility delivery at the community-level (OR: 0.04; 95% CI: 0.01-0.32). The interaction, depicted in Figure 1, shows that when husbands make decisions alone and community attitudes toward men’s support are low, the predicated probability of facility delivery is low. Conversely, when community attitudes toward men’s support are high, whether the husbands make decisions alone or not, the probability of facility delivery is higher and more similar to when the wife is involved in health care decisions.

**Figure 2:**
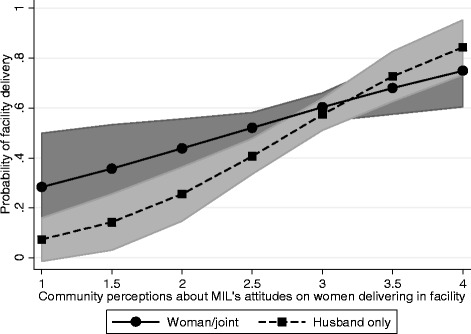
**Adjusted predictions at representative values with 95% confidence intervals for Model 6.** Community perceptions of mother-in-law’s attitudes toward facility delivery by decision-making autonomy.

Finally, Models 5 and 6 provide similar findings. Specifically, Model 5 shows that women who perceive that more mothers-in-law are supportive of facility delivery are more likely to deliver in a facility and women with low decision-making autonomy are less likely to deliver in a facility. Furthermore, controlling for women’s own attitudes toward mothers-in-law’s support for facility delivery, women living in communities where there is greater perceived support for facility delivery by mothers-in-law are more likely to deliver in a facility than all others (OR: 3.46; 95% CI: 1.85-6.45). Model 6 includes the interaction between women’s decision-making autonomy and mother-in-law’s community-level attitudes. The interaction, depicted in Figure 2, demonstrates that women who have low decision-making autonomy (husbands make decisions alone) have a lower predicted probability of a facility delivery when community attitudes toward mothers-in-law’s support for facility delivery are low. When community attitudes toward mothers-in-law’s support are higher, the effect of lower decision-making autonomy is more similar to when women have higher decision-making autonomy.

## Discussion

Using these unique data, we find that supportive community-level attitudes are associated with greater odds of facility delivery, even after controlling for a woman’s own perceptions about community attitudes. In addition, women with lower decision-making autonomy regarding their own health care have lower odds of facility delivery compared to women who are involved in health care decisions. In models that include community-level attitudes about men’s and mothers-in-law’s support for facility delivery, the interaction between autonomy and community attitudes is significant indicating that women with low decision-making autonomy who also live in communities that are less supportive of facility delivery are the least likely to have a facility delivery. Conversely, women who have low decision-making autonomy who live in communities that are more supportive of facility delivery are more similar to women with high decision-making autonomy. Therefore, perceived men’s and mothers-in-law’s community attitudes are influential, even for the least empowered women.

Previous studies have also demonstrated that there are psychological and social barriers to maternal and child health services [3,10,17]. These studies have generally focused on qualitative data whereas our study uses quantitative data to demonstrate similar influences. Our findings are consistent with other studies from Ghana that used Demographic and Health Survey data in terms of demographic characteristics associated with facility delivery [7,8]. However, our study is unique because of its focus on maternal and child health care utilization with specific questions on attitudes toward facility delivery at multiple levels (including perceived attitudes of husband’s and mothers-in-law).

Our findings corroborated findings from two previous studies that have shown that support for facility deliveries by mothers-in-law and husbands is associated with institutional delivery [27,28]. White and colleagues [28] found similar results in that women in rural Mali whose mothers-in-law agreed with traditional and cultural practices were less likely to deliver in a health facility. The traditional views of mothers-in-law may contradict more contemporary views of pregnancy and childbirth. Contrary to our results, rural Malian women’s own perceptions of delivery practices of women in the community, and the perceptions of their husbands, were not associated with place of delivery. Furthermore, Danforth and colleagues [27] found that spousal agreement about the place of delivery was associated with facility delivery in rural Tanzania. This evidence supports our notion that the opinions of both partners are important when deciding where to deliver their child.

Previous studies that have explored community-level attitudes about facility delivery did not have the same amount of detail that our study provides. Kruk and colleagues [26] showed that women’s perceptions of the importance of facility delivery at the village level were associated with facility delivery, controlling for women’s individual perceptions. However, they did not provide information on the perceptions of other influential people in the woman’s social network, including her husband and her mother-in-law. Stephenson and colleagues [25] used community-level variables to predict facility delivery in six African countries, but the variables they used were proxies for community norms and gender dynamics. For example, they used female educational attainment and level of facility delivery to approximate social norms related to the power of women and facility delivery, respectively. Our study builds upon these results by using indicators that provide a more direct assessment of perceived community norms and women’s decision-making autonomy.

There have been mixed results related to the association between women’s decision-making autonomy and the use of maternal and child health services. Studies on women’s decision-making power have shown associations between higher decision-making autonomy and increased antenatal care use [23,30], having a normal body mass index [35], having a birth preparedness plan [36], childhood immunization [23], and sick child care [35]. However, the linkage between women’s decision-making autonomy and facility delivery in sub-Saharan Africa is less commonly studied and has shown mixed results [29,30]. In a study using the 2008 Ghana Demographic and Health Survey, Moyer and colleagues [3] found that women who did not participate in decision-making regarding their own health care were less likely to deliver in a health facility. However, when other factors were considered, such as maternal literacy, health insurance coverage, and wealth, the association between women’s decision-making autonomy and facility delivery was no longer significant. When controlling for perceptions of mothers-in-law and husbands (Table 3, Models 3–6), we find that women who have lower decision-making autonomy about their own health care have lower odds of facility delivery compared to women who are involved in health care decisions. This suggests that there is something unique about household dynamics and spousal communication, beyond maternal education and household wealth, in these two districts in Ghana. However, when controlling for perceptions of other women, decision-making by someone other than the woman or her spouse was significantly related to facility delivery (Table 3, Model 1). Women in this decision-making scenario need to be examined more closely as this was a rare response with a large confidence interval.

The relationship between women’s decision-making autonomy and facility delivery is even more important when the attitudes and beliefs in the community in which women live are considered (Figures 1 and 2). The association between community norms and facility delivery is greater (i.e., the slope is steeper) among women whose husbands make their health care decisions compared to women who are involved in health care decisions about their own health. This has important implications for the importance of community norms and how they interact with household gender dynamics. Communities that support contemporary delivery practices may also be more supportive of gender equity with regards to household decision-making.

This study is not without limitations. First, this is a cross-sectional study and therefore it is not possible to determine the direction of causality. Possibly, women who deliver in a health facility become more favorable toward facility delivery after the fact rather than their attitudes toward facility delivery influencing their delivery behaviors. Therefore, we are only able to show associations with the available data. To better understand if attitudes influence behaviors, it would be necessary to have longitudinal data on women’s attitudes toward facility delivery prior to any pregnancy experience. The second limitation with these data is that we are using perceived attitudes of men and mothers-in-law. Unfortunately, data from these influential individuals were not collected in this study. That said, there is prior research that shows that perceptions of attitudes and norms are important to understanding health behaviors [37] and might matter more than actual attitudes in influencing health behaviors [38]. Third, responses of women’s attitudes and their perceptions of their husbands and mothers-in-law were correlated which meant that we were not able to determine if one is more influential than the other. Finally, given our use of quantitative data, it is not possible to answer the questions of “why” and “how” autonomy and community-level attitudes influence behaviors. This can be explored with qualitative data that goes into depth on decision-making autonomy and community attitudes simultaneously.

Future studies of barriers to women’s use of facility delivery in Ghana and elsewhere in sub-Saharan Africa should consider the role of decision-making autonomy and community norms. To do this correctly will require collecting data from multiple players (e.g., women, partners, mothers-in-law, and health care providers) as well as collecting data longitudinally to better understand the multiple influences. In particular, over time, women’s attitudes may change as well as community attitudes. Following communities and women longitudinally will permit a determination of which of these are the most important for future program development.

## Conclusions

The findings from this study are useful for the Maternal and Newborn Referrals Project of Project Fives Alive, and other similar maternal and child health programs, to inform future strategies to increase the use of institutional (or skilled) delivery. First, programs should continue to involve influential players, including husbands and mothers-in-law, and not just pregnant women. Notably, since the time these data were collected, the Maternal and Newborn Referrals Project of Project Fives Alive has tested strategies to engage husbands and mothers-in-law. This has been done through community engagement and mobilization led by traditional birth attendants, health staff, and quality improvement project staff. Community conversations involve influential individuals and encourage discussion on the importance of men attending antenatal and delivery care and the importance of delivering in a health facility. The final evaluation will permit an assessment of whether women (and their network members) who were exposed to these types of interventions were more likely to deliver in a health facility compared to women who were not exposed to these interventions. Second, programs that undertake community outreach activities to change community norms are likely to be more effective than programs that simply target individuals. By addressing community norms related to institutional delivery, especially birth location preferences among women and men, programs have the potential to help families become better prepared for obstetric emergencies should complications arise [39]. With these types of interventions, community norms should become more favorable toward facility delivery and women will be more likely to deliver in safer settings, even if they do not have the decision-making authority. Overall, maternal and child health programs that involve influential individuals and communities are likely to be the most successful at helping Ghana and other sub-Saharan African countries to attain their Millennium Development Goals for women’s and children’s health.
